# Platelet Factor 4 Attenuates Experimental Acute Liver Injury in Mice

**DOI:** 10.3389/fphys.2019.00326

**Published:** 2019-03-26

**Authors:** Hannah K. Drescher, Elisa F. Brandt, Petra Fischer, Stephan Dreschers, Reto A. Schwendener, M. Anna Kowalska, Ali Canbay, Hermann E. Wasmuth, Ralf Weiskirchen, Christian Trautwein, Marie-Luise Berres, Daniela C. Kroy, Hacer Sahin

**Affiliations:** ^1^Department of Internal Medicine III, University Hospital, RWTH Aachen, Aachen, Germany; ^2^Department of Neonatology, University Hospital, RWTH Aachen, Aachen, Germany; ^3^Institute of Molecular Cancer Research, University of Zurich, Zurich, Switzerland; ^4^Department of Pediatrics, Children’s Hospital of Philadelphia, Philadelphia, PA, United States; ^5^Institute of Medical Biology, Polish Academy of Sciences, Łódź, Poland; ^6^Department of Gastroenterology, Hepatology and Infectious Diseases, Otto von Guericke University of Magdeburg, Magdeburg, Germany; ^7^Institute of Molecular Pathobiochemistry, Experimental Gene Therapy, and Clinical Chemistry, University Hospital, RWTH Aachen, Aachen, Germany

**Keywords:** hepatoprotective effects, PF4, liver, acute liver damage, macrophages, activated protein C

## Abstract

Platelet factor 4 (PF4) is a pleiotropic inflammatory chemokine, which has been implicated in various inflammatory disorders including liver fibrosis. However, its role in acute liver diseases has not yet been elucidated. Here we describe an unexpected, anti-inflammatory role of PF4. Serum concentrations of PF4 were measured in patients and mice with acute liver diseases. Acute liver injury in mice was induced either by carbon tetrachloride or by D-galactosamine hydrochloride and lipopolysaccharide. Serum levels of PF4 were decreased in patients and mice with acute liver diseases. *PF4^-/-^* mice displayed increased liver damage in both models compared to control which was associated with increased apoptosis of hepatocytes and an enhanced pro-inflammatory response of liver macrophages. In this experimental setting, *PF4^-/-^* mice were unable to generate activated Protein C (APC), a protein with anti-inflammatory activities on monocytes/macrophages. *In vitro*, PF4 limited the activation of liver resident macrophages. Hence, the systemic application of PF4 led to a strong amelioration of experimental liver injury. Along with reduced liver injury, PF4 improved the severity of the pro-inflammatory response of liver macrophages and induced increased levels of APC. PF4 has a yet unidentified direct anti-inflammatory effect in two models of acute liver injury. Thus, attenuation of acute liver injury by systemic administration of PF4 might offer a novel therapeutic approach for acute liver diseases.

## Introduction

Acute and chronic liver diseases are a common health burden worldwide. Acute liver failure (ALF) is defined as the sudden loss of liver metabolic function ultimately resulting in coagulopathy and encephalopathy most often induced by alcoholic hepatitis, viral hepatitis or drug-induced injury (Garcia-Tsao, 2018). Its appearance is associated with high patient mortality and morbidity worldwide, in spite of significant progress in liver support systems and liver transplantation (O’Grady, 2014). Several studies have been conducted to understand the mechanisms of acute liver diseases and to search for alternative therapies. One of the common understandings is that upon direct or indirect activation by endogenous (e.g., bacterial endotoxic LPS) or exogenous (e.g., drugs and toxins) noxae, liver resident macrophages (Kupffer cells) and infiltrating monocytes/macrophages secrete high amounts of various cytokines, including IL-1, IL-6, IL-12 and TNF-α, thereby mediating excessive hepatocyte death leading to the recruitment of immune cells (Groeneveld et al., 1988; Gantner et al., 1995; Rizzardini et al., 1998; Sato et al., 2014). Thus, platelets are among the first cells, which are actively recruited to the liver, where they perpetuate organ damage (Yamaguchi et al., 2006; Yu et al., 2011; Miyakawa et al., 2015). Besides hemostasis and thrombosis, platelets are intrinsically involved in hepatic inflammation and immune responses (Iannacone et al., 2005; Lang et al., 2008). However, apart from serotonin (Lang et al., 2008), the role of other platelet-derived inflammatory mediators including chemokines remains obscure in acute liver diseases.

Upon adhesion and activation, platelets rapidly release a wide range of chemokines from their α-granules (von Hundelshausen et al., 2007). Among these, PF4 is the most abundant. It has been shown to modulate the immune system by promoting recruitment, survival and differentiation of innate and adaptive immune cells, e.g., monocytes/macrophages (Scheuerer et al., 2000; Koenen et al., 2009; Gerdes et al., 2011; Hartwig et al., 2014). Moreover, PF4 binds to other chemokines such as IL-8 (Dudek et al., 2003) and RANTES (Koenen et al., 2009) and amplifies their pro-inflammatory effects. Notably, PF4 has already been designated as an inflammatory mediator in atherosclerosis (Koenen et al., 2009) and liver fibrosis (Zaldivar et al., 2010). Moreover, serum concentrations and the intrahepatic expression of PF4 were elevated in patients with viral hepatitis and non-alcoholic steatohepatitis (Zaldivar et al., 2010), indicating its potential involvement in chronic liver diseases.

In contrast to these data and the general appreciation of a pro-inflammatory effect of PF4 function, recent findings demonstrate that this chemokine also exerts immune-protective effects under certain pathological circumstances. These studies have identified PF4 as a negative regulator of a functionally relevant Th17 response in different transplant models (Shi et al., 2014; Guo et al., 2015). However, the role of PF4 in acute liver diseases has not yet been delineated. Here, we describe a previously unexplored and unexpected role of the platelet-derived chemokine PF4 in human and experimental acute liver diseases. Accordingly, we show that PF4 is decreased in human acute liver failure (ALF) and plays a functional role in experimental acute liver diseases by impairing activation of liver resident macrophages.

## Materials and Methods

### Serum Samples From Patients With Acute Liver Failure

Overall, PF4 and APC concentrations were measured in serum of 22 patients with ALF and 12 healthy subjects using a human ELISA Kit (R&D Systems and Biozol, respectively) following the manufacturer’s instructions. The demographic and clinical data of these patients are given in Supplementary Table 1. The study has been approved by the local ethics committee and all participants have granted informed consent prior to inclusion.

### Murine *in vivo* Experiments

Male C57BL/6 wild-type mice (6–8 weeks) were purchased from Charles River Laboratories. PF4^-/-^ animals with a target deletion of the PF4 gene were established on the C57BL/6 background as described (Sachais et al., 2007) and backcrossed for more than 10 generations. All *in vivo* experiments were performed after approval by the Animals Welfare Review Board and by the German legal authorities of North-Rhine Westfalia (LANUV). Acute liver injury was induced either by a single intraperitoneal injection of D-GalN (700 mg/kg body weight, Roth) and LPS (10 μg/kg body weight, Sigma) for 2 and 6 h or by a single intraperitoneal injection of CCl_4_ (0.6 mL/kg body weight, Merck) for 24 h following standard protocols (Hamesch et al., 2015; Scholten et al., 2015). For macrophage depletion, liposome-encapsulated clodronate, termed clodrolip, and control liposomes were injected intraperitoneally at 100 mg per 1 kg body weight, 24 h prior to GalN/LPS administration. In a separate experiment, *PF4*^-^*^/^*^-^ and wild-type mice were treated with recombinant mouse PF4 (1 mg/kg body weight, Biomol) parallel to GalN/LPS (Kowalska et al., 2011). Liver injury was histologically assessed by Hematoxylin Eosin (H&E) staining. Serum AST and ALT were measured as previously described (Zaldivar et al., 2012). Plasma concentrations of APC were measured using a mouse ELISA kit (Hölzel Biotech) following the manufacturer’s instructions.

### Quantification of Cell Death

TUNEL assay (Roche) was used to detect cell death histologically. The percentage of the TUNEL^+^ area was quantified in three independent fields per liver using the ImageJ software as described before (Zaldivar et al., 2012).

### Analysis of Liver Protein and Gene Expression

Isolation of total hepatic protein and RNA from snap-frozen liver tissue samples was performed as previously described (Berres et al., 2010). After SDS gel electrophoresis and wet blotting of total protein, cleaved caspase-3 protein was detected by a rabbit anti-mouse cleaved caspase-3 antibody (Cell Signaling) followed by horseradish peroxidise-conjugated anti-rabbit IgG (DAKO) and ECL substrate (Thermo Fisher Scientific). β-Actin was used as loading control. Intrahepatic and serum PF4 concentrations were determined using a murine ELISA kit (R&D Systems) following the manufacturer’s instructions. Quantitative RT-PCR (RT-qPCR) was carried out for TGF-β, TNF-α, IL-6, IL-10, and KC with Assays-on-Demand (Applied Biosystems). β-Actin was used as reference gene.

### Histological Quantification of Immune Cells

Five-μm frozen liver tissue sections were fixed and stained with F4/80, CD11b (both obtained from AbD Serotec), Ly6G (Thermo Fisher Scientific) and CD41 (BD Biosciences) antibodies followed by a suitable secondary antibody conjugated with Alexa fluor^®^ 488 or Cy3 (both Thermo Fisher Scientific). Cell nuclei were counterstained with DAPI (Vector Laboratories). The percentage of the stained area was quantified in three independent magnification fields per mouse (Berres et al., 2010).

### Flow Cytometry Analysis of Hepatic Immune Cells

For flow cytometry analysis, leukocytes from harvested livers were isolated by enzymatic digestion. After density gradient centrifugation and washing, these cells were stained with fluorochrome-conjugated antibodies for CD45 (BD Biosciences), CD3, CD8, CD4, and NK1.1 antibodies (all from eBioscience). Stained cells were analyzed using a LSR Fortessa flow cytometry system (BD Biosciences) and data was quantified using FlowJo (TreeStar) (Berres et al., 2010; Zaldivar et al., 2010).

### *In vitro* Experiments

Murine primary Kupffer cells were isolated from livers of wild-type mice by collagenase digestion followed by density gradient centrifugation as described by Taura et al. (2008). Cells were purified by MACS magnetic bead separation for F4/80 (Miltenyi Biotec.). For chemokine stimulation, cells were starved in RPMI containing 0.5% FCS (starving medium) for 16 h and stimulated with LPS (500 ng/mL, Sigma) in the presence or absence of recombinant mouse PF4 (1 μM, R&D Systems) for 6 h.

### Analysis of Macrophage Phagocytosis

To quantify the phagocytic response of Kupffer cells, *E. coli* expressing the red fluorescent protein DsRed were used (Berger et al., 2010). To this end, Kupffer cells (1 × 10^6^) were stimulated for 6 h with LPS and co-stimulated with PF4 as mentioned. Two hours before harvesting, these cells were challenged with 1 × 10^7^ DsRed-expressing *E. coli*. After staining with F4/80 antibody (BD Biosciences), phagocytic Kupffer cells were measured on a BD FACSCanto II system (BD Biosciences).

### Analysis of Supernatant of Kupffer Cells

Upon isolation, the Kupffer cells were separated by MACS magnetic beads separation (F4/80, Miltenyi Biotec) (Wu et al., 2012) and exposed to LPS and PF4 for 6 h. The supernatant was harvested and TNF-α was measured using a mouse ELISA Kit (R&D Systems) following the manufacturer’s instructions. Nitric oxide was determined using a method with Vanadium(III) chloride reduction followed by the Griess reagent system (Promega) (Egan et al., 2002). Briefly, Vanadium(III) chloride reduced the sample nitrate (NO_3_) to NO_2_ which thereby reacted with the Griess reagent, sulphanilamide, and *N*-(1-naphthyl)-ethylenediamine. The absorbance was then measured photometrically at 540 nm.

### Quantification of PF4 Expression in Kupffer Cells

Isolated and MACS-separated Kupffer cells were used to assess the PF4 expression. To this end, these cells were stimulated with LPS for 6 h and collected in Cytospin Collection Fluid (Thermo Fisher Scientific). 10^5^ cells were cytocentrifuged with Cytospin (Thermo Fisher Scientific) and then stained with PF4 and DAPI. RT-qPCR was performed for PF4 with Assays-on-Demand (Applied Biosystems). β-Actin was used as reference gene.

### Statistical Analysis

Statistical quantification if two groups were compared was performed with the Students unpaired *t*-test. Results were described as mean ± SEM. When comparing more groups, ANOVA testing with Turkey’s multiple comparison post-test was performed. At least five animals per group were analyzed. Outliers were excluded, when Grubb’s test calculated on graph pad homepage^1^ calculated a significant outlier. All statistical tests were performed using Graph-Pad Prism^®^. A *p*-value of < 0.05 was defined as significant (^∗^*p* < 0.05, ^∗∗^*p* < 0.01, ^∗∗∗^*p* < 0.001).

## Results

### Serum PF4 Is Decreased in Human and Experimental Acute Liver Diseases

**Figure 1:**
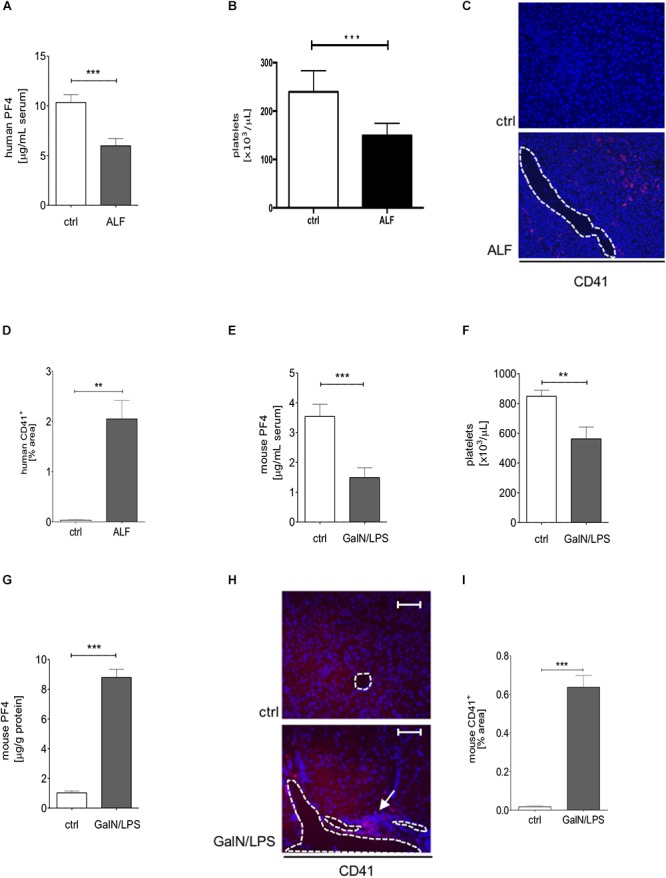
Serum PF4 is decreased in human patient samples and mice with acute liver disease. Serum levels of PF4 were decreased in patients with acute liver failure (ALF) when compared to healthy controls (ctrl) assessed by ELISA **(A)**. In line, platelet numbers were decreased in humane blood samples **(B)**. Immunofluorescent staining for CD41^+^ cells (CD41 (red), nuclei were counterstained with Dapi (blue), vessels are marked with white lines, magnification x200) **(C)**. The quantitative evaluation shows that the platelet content within the liver of patients with ALF was increased compared to control (ctrl) **(D).** Comparable to the human situation, wild-type mice treated with GalN/LPS had reduced PF4 levels in serum **(E)** and decreased numbers of platelets in blood **(F)**. Contrarily, the intrahepatic protein expression of PF4 was significantly increased when compared to control (ctrl) **(G)**. This alteration was associated by augmented accumulation of platelets within the liver of wild-type mice (CD41 (red), Nuclei were counterstained with Dapi (blue), vessels are marked with white lines, magnification x200) **(H)** also shown in the quantitative analysis of the staining **(I)**. *n* = 8, ^∗∗^*p* < 0.01, ^∗∗∗^*p* < 0.001.

To investigate the expression of PF4 in human ALF, we investigated the PF4 concentration in serum samples in respective patients and healthy controls. PF4 serum levels and platelet numbers were decreased in patients with ALF when compared to healthy individuals which was markedly linked to an increased accumulation of platelets within the liver (Figure 1A–D). The human data was confirmed in wild-type mice treated with GalN/LPS for 6 h to induce acute liver injury, showing decreased PF4 concentrations (Figure 1E). We next assessed whether these decreases in PF4 serum levels were also reflected by reduced numbers of platelets in blood. Indeed, platelets were significantly reduced after GalN/LPS treatment compared to controls (Figure 1F), supporting recent findings from mouse and human studies (Schiodt et al., 2003; Miyakawa et al., 2015). Notably, intrahepatic PF4 protein expression was significantly increased in GalN/LPS-treated wild-type mice compared to controls (Figure 1G). This alteration was associated with an enhanced accumulation of platelets within the liver (Figure 1H,I).

### PF4-Deficient Mice Display Severe Liver Injury After GalN/LPS Treatment

**Figure 2:**
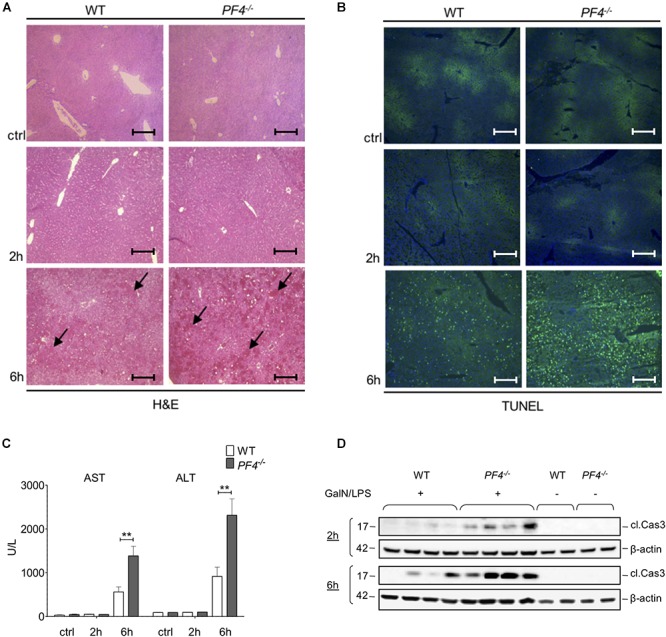
Deletion of PF4 is associated with severe acute liver injury after GalN/LPS challenge. Liver injury was assessed by H&E staining (black arrows exemplary point at necrotic areas, magnification x100) **(A)** and TUNEL assay (TUNEL^+^ cells (green), nuclei were counterstained with Dapi (blue), magnification x100) **(B)**. The administration of GalN/LPS for 6 h led to enlarged areas of liver damage and a significantly increased number of TUNEL^+^ cells within the liver of *PF4^-/-^* mice compared to their wild-type counterparts. These alterations were confirmed by significantly increased levels of serum transaminases AST and ALT in *PF4^-/-^* mice **(C)**. Severe liver injury in *PF4^-/-^* mice was associated with augmented activation of Caspase-3 2 and 6 h after treatment as assessed by Western blot. β-Actin was used to demonstrate equal protein loading **(D)**. *n* = 8, ^∗∗^*p* < 0.01.

Next, we investigated the functional relevance of PF4 in experimental acute liver injury. *PF4^-/-^* and wild-type mice were treated with GalN/LPS for 2 and 6 h. Compared to their wild-type counterparts, *PF4^-/-^* mice displayed increased parenchymal damage associated with tissue necrosis and hemorrhage within 6 h (Figure 2A). Analysis of apoptosis via TUNEL Assay confirmed increased liver injury in *PF4^-/-^* mice (Figure 2B). These mice showed more TUNEL^+^ cells 6 h after GalN/LPS challenge (Figure 2B and Supplementary Figure 1A). These changes were reflected by significantly increased serum AST and ALT levels after 6 h (Figure 2C). However, analysis of liver injury 2 h after treatment showed no obvious differences between untreated and treated mice and also between mouse strains (Figure 2A–C). Apoptosis is a crucial hallmark of GalN/LPS-induced hepatitis (Leist et al., 1995), we thus investigated intrahepatic caspase-3 activation, serving as an important key effector of apoptosis, in GalN/LPS-treated mice by Western blot analysis. In wild-type mice GalN/LPS treatment resulted in caspase-3 activation at early stages of liver injury (Figure 2D) which was further enhanced 6 h after treatment (Figure 2D). In contrast, caspase-3 activation was significantly stronger increased in *PF4^-/^* mice 2 and 6 h after GalN/LPS injection (Figure 2D and Supplementary Figure 1B), suggesting a direct link between PF4 and the degree of apoptotic cell death in the liver. Collectively, these unexpected data show that genetic deletion of *PF4* enhances GalN/LPS-induced liver damage, despite its known role as a pro-inflammatory chemokine (Deuel et al., 1981; Scheuerer et al., 2000).

### Deletion of PF4 Is Associated With a Stronger Pro-inflammatory Immune Response in the GalN/LPS Model

**Figure 3:**
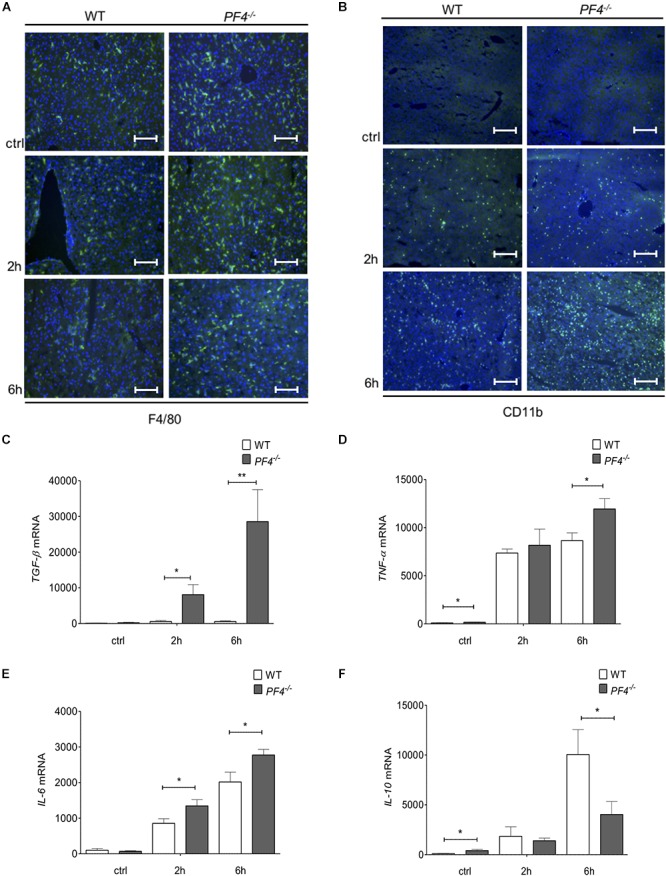
*PF4^-/-^* mice displayed increased pro-inflammatory conditions in the GalN/LPS model. The genetic deletion of PF4 in mice was associated with an increased number of intrahepatic F4/80^+^ (F4/80^+^ cells (green), nuclei were counterstained with Dapi (blue), magnification x200) **(A)** and CD11b^+^ immune cells (CD11b^+^ cells (green), nuclei were counterstained with Dapi (blue), magnification x100) **(B)** 6 h after GalN/LPS treatment when compared to wild-type mice. The increased frequency of liver macrophages in *PF4^-/-^* mice was further confirmed by a time-dependent increase of *TGF*-β **(C)**, *TNF-α* **(D)** and *IL-6* **(E)** and decrease of IL-10 **(F)** mRNA expression. *n* = 8, ^∗^*p* < 0.05, ^∗∗^*p* < 0.01.

To further investigate the differences in disease progression between *PF4^-/-^* and wild-type mice, we examined the composition of cellular infiltrates within the liver. Untreated (ctrl) and GalN/LPS treated *PF4^-/-^* animals showed no differences in the frequency of F4/80^+^ and CD11b^+^ immune cells compared to their wild-type counterparts after 2 h treatment. In contrast, *PF4^-/-^* mice treated for 6 h accumulated significantly more F4/80^+^ and CD11b^+^ macrophages within the liver (Figure 3A,B and Supplementary Figures 1C,D). Detailed characterization of F4/80^+^ cells showed a significant increase of CD11b^+^/F4/80^low^ infiltrating macrophages in *PF4^-/-^* mice after 6h GalN/LPS treatment compared to wild-type. CD11b^+^/F4/80^high^ remained unchainged (Supplementary Figure 1E). Consistent with macrophage frequency, the intrahepatic mRNA expression of the inflammatory cytokines encoding for TGF-β, TNF-α and IL-6 were significantly increased in *PF4^-^*^/^*^-^* mice compared to wild-type mice (Figure 3C–E). Conversely, the mRNA expression of *IL-10*, a cytokine with anti-inflammatory activities (Chaudhry et al., 2011), was reduced in *PF4^-/-^* mice (Figure 3F). Of note, genetic deletion of *PF4* in mice during steady-state led to low, but significantly increased mRNA levels of *TNF-α* and *IL-10* when compared to wild-type mice (Figure 3D,F), suggesting that this chemokine is important for liver macrophage homeostasis. Progression of acute liver injury is characterized by an increase of mediators facilitating the infiltration of different immune cells types, including neutrophils and T cells (34). Indeed, mRNA expression of the neutrophil chemoattractant *KC* (murine IL-8) was significantly altered in *PF4^-^*^/^*^-^* compared to wild-type mice (Supplementary Figure 2A). Functionally, increased *KC* mRNA expression was associated with severe neutrophil infiltration to the liver of *PF4^-/-^* mice (Supplementary Figure 2B), arguing against a major role of PF4 in attracting neutrophils (Deuel et al., 1981; Zaldivar et al., 2010). However, analysis of intrahepatic T cell subpopulations by flow cytometry revealed no obvious differences in frequency and absolute cell numbers of CD4^+^ and CD8^+^ T cells between *PF4^-/-^* and wild-type animals (Supplementary Figure 3). Overall, these findings suggest that liver resident F4/80^+^ macrophages play a functional role in the phenotype observed in our model.

### Deletion of PF4 Aggravates Severity of Liver Injury in the CCl_4_ Model of Acute Liver Injury

After having characterized the significant impact of PF4 on disease progression in the GalN/LPS model, we analyzed whether this finding could be confirmed in another acute toxic model. We tested the effect of PF4 on disease progression 24 h after a single CCl_4_ injection. *PF4^-/-^* mice displayed a strong increase in liver damage after CCl_4_ treatment compared to wild-type mice assessed by the analysis of H&E stained liver sections (Supplementary Figure 4A). This finding was further strengthened by a significant increase of TUNEL^+^ cells and the inflammation-associated genes *TGF*-β and *TNF-α* within *PF4^-/-^* livers (Supplementary Figures 4B–D).

### Absence of PF4 Is Associated With Reduced APC Generation

**Figure 4:**
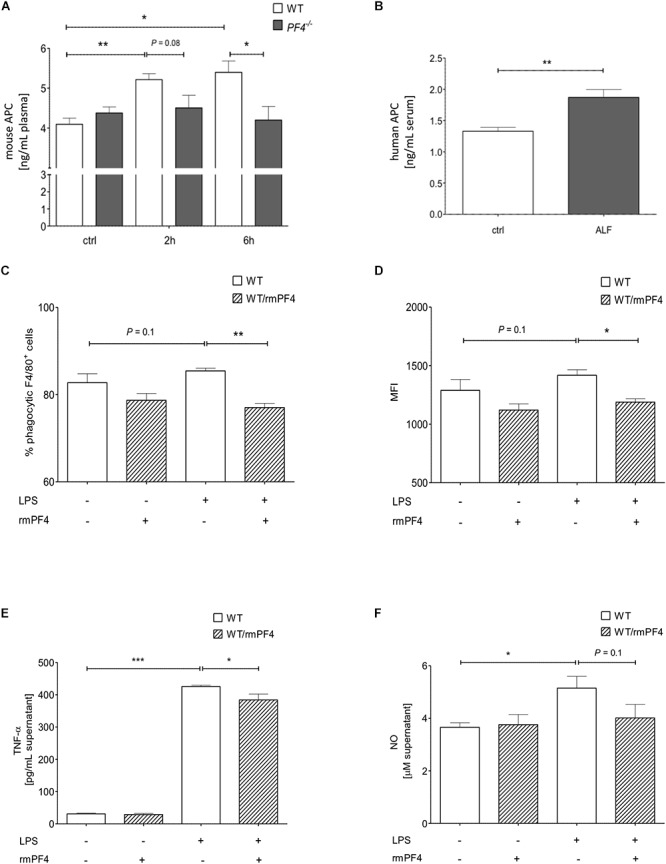
Platelet factor 4 impairs Kupffer cell activation *in vitro. PF4^-/-^* mice were deficient in generating APC 2 and 6 h after GalN/LPS treatment when compared to wild-type mice **(A)**. Serum levels of human APC were also increased in patients with ALF when compared to control (ctrl) as assessed by ELISA **(B)**. *n* = 8, ^∗^*p* < 0.05, ^∗∗^*p* < 0.01. Recombinant mouse PF4 (rmPF4) counteracted LPS-mediated phagocytic response of Kupffer cells **(C,D)**. Furthermore, PF4 inhibited the LPS-induced TNF-α **(E)** and NO release **(F)** by Kupffer cells into the supernatant. *n* = 6, ^∗^*p* < 0.05, ^∗∗^*p* < 0.01, ^∗∗∗^*p <* 0.001.

Platelet factor 4 is known to promote recruitment and pro-inflammatory differentiation of monocyte-derived macrophages (Scheuerer et al., 2000). We next wanted to test whether PF4 exerts anti-inflammatory effects on liver infiltrating macrophages in our model. Recently, Kowalska et al. (2007) could show that endogenous PF4 stimulates APC generation *in vivo*. Notably, APC has been shown to directly inhibit activation and migration of monocytes as well as TNF-α production of macrophages, thereby reducing endotoxemia and sepsis mortality (Stephenson et al., 2006; Kerschen et al., 2007; Cao et al., 2010). Thus, we next analyzed APC concentrations in plasma of *PF4^-/-^* and wild-type mice by ELISA. We found a time-dependent increase of APC generation in wild-type mice 2 and 6 h after GalN/LPS challenge, which was completely blunted in *PF4^-/-^* mice (Figure 4A). In patients with ALF, serum levels of APC were also enhanced compared to healthy controls (Figure 4B). These data suggest that PF4 has the potential to suppress the pro-inflammatory response of macrophages *via* APC.

### PF4 Impairs Activation of Kupffer Cells*in vitro*

**Figure 5:**
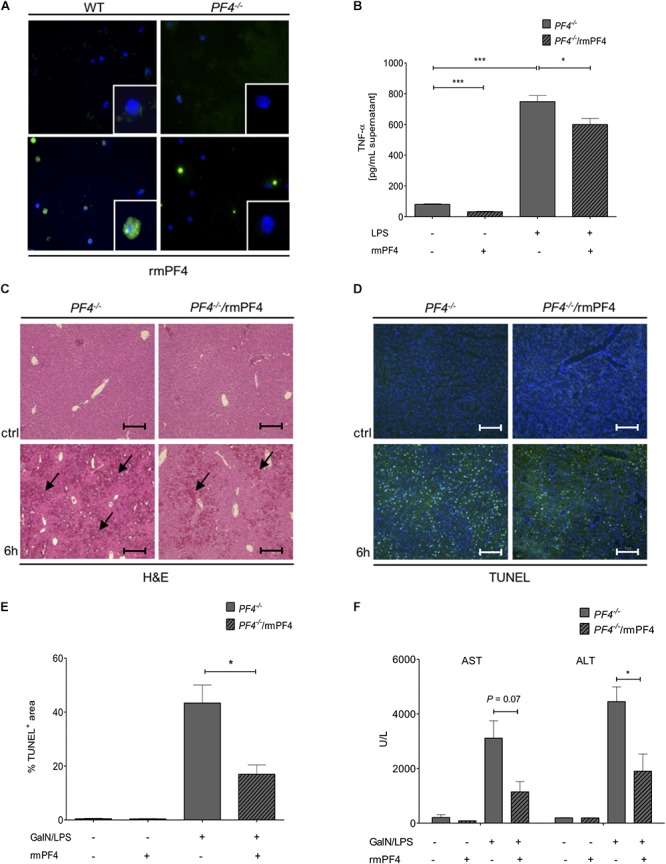
Systemic PF4 abrogated the GalN/LPS-induced liver injury in *PF4^-/-^* mice. Kupffer cells were also a source of LPS-stimulated PF4 as assessed by immunofluorescence staining (PF4 (green), nuclei were counterstained with Dapi (blue), magnification x600) **(A)**. The stimulation of *PF4^-/-^* Kupffer cells with PF4 reduced the basal as well as the LPS-induced TNF-α protein level in supernatant **(B)**. The response of recombinant mouse PF4 (rmPF4) was analyzed in the GalN/LPS model by H&E staining (black arrows exemplary point at necrotic areas, magnification x100) **(C)** and TUNEL assay (TUNEL^+^ cells (green), nuclei were counterstained with Dapi (blue), magnification x100) **(D,E)**. The protective effect of PF4 was further reflected by reduced serum levels of transaminases **(F)**. *n* = 6, ^∗^*p* < 0.05, ^∗∗∗^*p* < 0.001.

The *in vivo* results suggested a functional association between the chemokine PF4 and a reduced pro-inflammatory response of macrophages. Among monocytes/macrophages, liver resident macrophages (i.e., Kupffer cells) are crucial mediators of GalN/LPS-induced hepatitis (Antoniades et al., 2008). Thus, we next assessed whether PF4 directly affects Kupffer cell biology *in vitro*. Three parameters were tested: phagocytosis, TNF-α and nitrit oxide (NO) release. Phagocytosis of DsRed-expressing *E. coli* was induced by LPS in primary wild-type Kupffer cell cultures (Supplementary Figure 5A). As depicted in Figure 4C,D, PF4 significantly abrogated the phagocytic response of LPS on these cells. Moreover, PF4 attenuated the LPS-induced TNF-α expression and NO release by Kupffer cells into the supernatant (Figure 4E,F). This data confirmed our *in vivo* results showing that PF4 inhibits activation of tissue macrophages. Since tissue resident macrophages express PF4 (de Jong et al., 2008; Yeo et al., 2016), we tested whether primary isolated Kupffer cells express PF4. We confirmed the expression of PF4 in primary wild-type Kupffer cells by RT-qPCR and immunofluorescence analysis. Upon stimulation by LPS, PF4 expression was further increased in Kupffer cells, whereas in *PF4^-/-^* Kupffer cells PF4 expression was absent (Figure 5A and Supplementary Figure 5B). Remarkably, stimulation of *PF4^-/-^* Kupffer cells with recombinant PF4 reduced its basal TNF-α release, indicating that PF4 might be a local autocrine signal maintaining Kupffer cell homeostasis. Parallel to wild-type Kupffer cells, PF4 also reduced the LPS-induced TNF-α release (Figure 5B).

### PF4 Administration Reversed GalN/LPS-Induced Liver Injury in PF4*^-^*^/^*^-^* Mice

**Figure 6:**
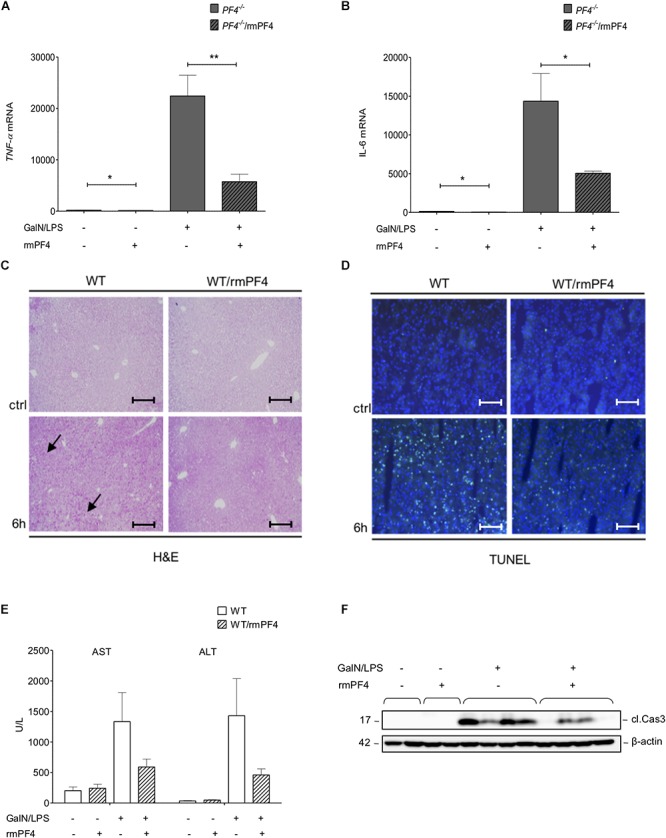
Attenuation of liver injury by systemic PF4 also in wild-type mice. Attenuated liver injury in *PF4^-/-^* animals after treatment with recombinant PF4 was further associated with decreased mRNA expression of *TNF-α* **(A)** and *IL-6* **(B)**. The therapeutic potential of PF4 was validated in the GalN/LPS model by H&E staining (black arrows exemplary point at necrotic areas, magnification x100) **(C)** and TUNEL assay (TUNEL^+^ cells (green), nuclei were counterstained with Dapi (blue), magnification x200) **(D)**. PF4 decreased the GalN/LPS induced liver damage. These results are supported by reduced serum levels of transaminases **(E)** and decreased activation of caspase-3 **(F)**. *n* = 6, ^∗^*p* < 0.05, ^∗∗^*p* < 0.01.

Given these *in vitro* findings, we postulated that administration of recombinant PF4 might reverse the phenotype of GalN/LPS treated PF4*^-^*^/^*^-^* mice. In these mice, injection of PF4 indeed abrogated the GalN/LPS-induced liver injury (Figure 5C) and hepatocellular death (Figure 5D,E). These functional effects of systemic PF4 also translated into reduced serum levels of AST and ALT after GalN/LPS administration (Figure 5F). This response was further confirmed by reduced hepatic mRNA levels of *TNF-α* and *IL-6* (Figure 6A,B). Importantly, the administration of PF4 led to a significant decrease of enhanced basal mRNA levels of *TNF-α* and *IL-6* in untreated *PF4^-/-^* animals, again suggesting a direct effect of PF4 on liver macrophage homeostasis.

### Therapy of Mice With PF4 Ameliorates GalN/LPS-Induced Liver Injury *in vivo*

To test whether systemic administration of PF4 can alter the progression of acute liver disease in wild-type mice, animals treated with GalN/LPS were additionally treated with recombinant PF4 for 6 h. Mice with recombinant PF4 showed a strong reduction in liver injury compared to vehicle-treated mice. This amelioration in disease progression gets evident when analyzing H&E and TUNEL stained liver sections (Figure 6C,D and Supplementary Figure 6A). In accordance with the results obtained in *PF4^-/-^* mice, serum levels of AST and ALT were significantly reduced by administration of PF4 compared to vehicle treatment (Figure 6E), findings that were confirmed by reduced caspase-3 activity (Figure 6F and Supplementary Figure 6B).

### PF4 Improves Severity of the Inflammatory Response in the GalN/LPS Model

**Figure 7:**
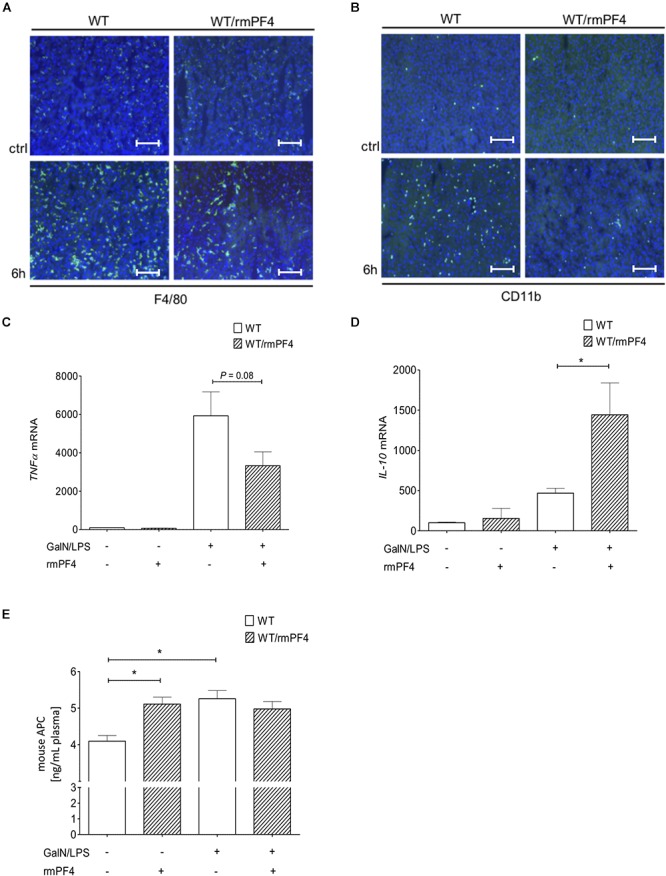
Administration of recombinant PF4 reduces GalN/LPS induced liver injury. PF4 therapy reduced the number of F4/80^+^ and CD11b^+^ immune cells *in vivo*. The numbers of F4/80^+^ (F4/80^+^ cells (green), nuclei were counterstained with Dapi (blue), magnification x200) **(A)** and CD11b^+^ (CD11b^+^ cells (green), nuclei were counterstained with Dapi (blue), magnification x200) **(B)** immune cells were reduced in PF4 treated wild-type mice when compared to vehicle treated mice. The reduced number of liver macrophages in PF4 treated wild-type mice was associated with a decreased mRNA expression of *TNF-α* **(C)** as well as increased mRNA expression of *IL-10* **(D)**. Exogenous PF4 also influenced APC generation in these mice **(E)**. *n* = 6, ^∗^*p* < 0.05.

Importantly, together with reduced liver injury, mice treated with PF4 showed a strong decrease in the infiltration of F4/80^+^ (Figure 7A and Supplementary Figure 6C) and CD11b^+^ (Figure 7B and Supplementary Figure 6D) macrophages when compared to vehicle-treated mice. These observed differences were associated with a strong reduction of *TNF-α* and also elevation of *IL-10* mRNA expression in PF4-treated mice (Figure 7C,D). However, PF4 treatment led to no differences in *KC* mRNA level and neutrophil influx (data not shown), suggesting that a main effect of PF4 *in vivo* is modulating macrophage activation which results in reduced apoptotic cell death in the liver. Based on our findings we next tested whether exogenous PF4 influences APC production. Notably, administration of PF4 or GalN/LPS alone led to a significant increase in APC levels in plasma of wild-type mice when compared to untreated animals (Figure 7E). However, the administration of PF4 concomitantly to GalN/LPS did not further increase this effect, suggesting that the dose-dependent high peak plasma concentration of APC was achieved in the earlier phase of liver injury (Kowalska et al., 2011).

### Depletion of Liver Macrophages Attenuates GalN/LPS-Induced Liver Injury in PF4*^-^*^/^*^-^* Mice

**Figure 8:**
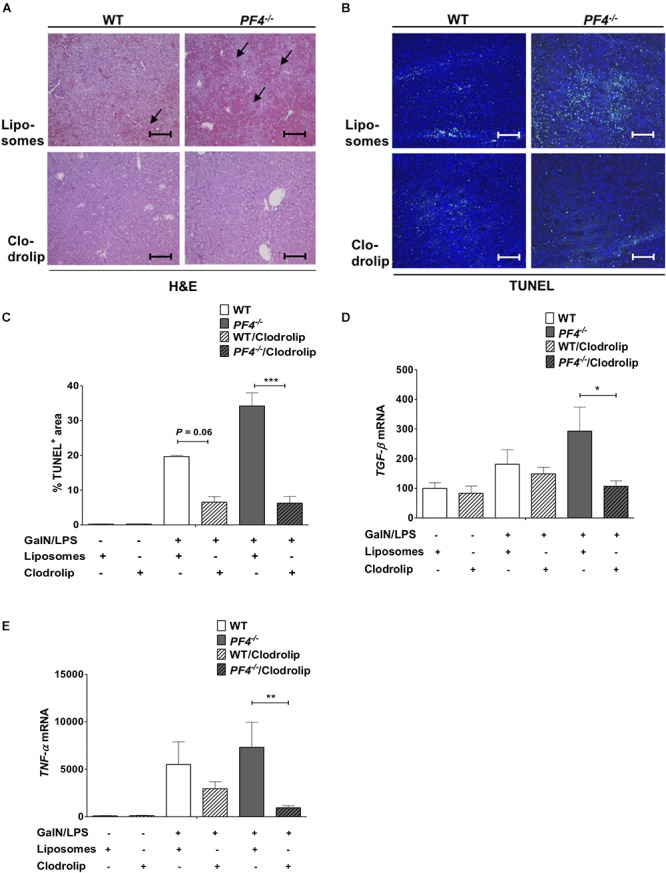
Depletion of macrophages reduced liver injury in *PF4^-/-^* mice. Macrophage depletion with clodrolip led to a reduced GalN/LPS-induced liver injury in WT mice compared to vehicle (liposomes) treated mice. These hepatoprotective effects were more pronounced in *PF4^-/-^* mice. Liver injury was determined by H&E staining (black arrows exemplary point at necrotic areas, magnification 100x) **(A)** and TUNEL assay (TUNEL^+^ cells (green), nuclei were counterstained with Dapi (blue), magnification 100x) **(B,C)** These results were supported by decreased TGF-β **(D)** and TNF-α **(E)** mRNA expression. *n* = 7, ^∗^*p* < 0.05, ^∗∗^*p* < 0.01, ^∗∗∗^*p <* 0.001.

To investigate the role of macrophages and macrophage derived PF4 in more detail we next depleted macrophages in WT and PF4*^-^*^/^*^-^* mice. To work out the impact on acute liver injury we performed additional GalN/LPS administration for 6 h. Deletion of F4/80^+^ macrophages in the liver could already be achieved by administration of a single dose clodrolip (Supplementary Figure 7). Depletion of these immune cells led to reduced liver injury in PF4*^-^*^/^*^-^* mice and also WT mice compared to animals injected with empty liposomes (Figure 8A). Reduced liver injury was further reflected by less intrahepatic TUNEL^+^ cells (Figure 8B,C) and decreased mRNA expression of TGF-β and TNF-α (Figure 8D,E). However, the hepato-protective effect of macrophage depletion was significantly more pronounced in PF4*^-^*^/^*^-^* mice compared to equally treated controls. Thus, we hypothesize that the increased pro-inflammatory response of macrophages in PF4*^-^*^/^*^-^* mice after administration of GalN/LPS is directly involved in increased liver damage observed in the PF4*^-^*^/^*^-^*.

## Discussion

In this study, we have uncovered an unexpected anti-inflammatory role of the chemokine PF4 in two independent mouse models of acute liver injury and provide first data for its regulation in patients with ALF. In patients and mice, serum PF4 concentrations were decreased compared to untreated controls which were associated with reduced platelet numbers in blood and increased levels of platelets and PF4 within the liver. Our findings extend previous human and mouse acute liver injury studies showing depletion of blood platelets and accumulation of platelets within the liver (Schiodt et al., 2003; Miyakawa et al., 2015). These observations suggest that decreased serum PF4 concentration and depletion of blood platelets are associated with progression of acute liver diseases.

However, the statistical association of PF4 serum levels and acute liver disease alone cannot imply a causal relationship. Thus, we analyzed the functional relevance of PF4 in different mouse models of experimental acute liver diseases. To this end, *PF4^-/-^* and wild-type mice were challenged with GalN/LPS or CCl_4_. Interestingly, despite the assumption that platelets and PF4 induce a pro-inflammatory immune response (Scheuerer et al., 2000; von Hundelshausen et al., 2007; Koenen et al., 2009), *PF4^-/-^* mice were more prone to liver injury when compared to equally treated wild-type mice.

Liver resident and infiltrating macrophages are crucial in the pathogenesis of acute liver injury (Tacke and Zimmermann, 2014). The analysis of the immune phenotype of *PF4^-/-^* mice after acute intoxication revealed an increased intrahepatic frequency of F4/80^+^ and CD11b^+^ immune cells, together with an increased mRNA expression of pro-inflammatory macrophage cytokines. Among these, TNF-α is central in the perpetuation of liver injury mainly by inducing apoptosis of hepatocytes (Leist et al., 1995). We in fact found elevated activation of caspase-3, a key effector of apoptosis, in the liver of *PF4^-/-^* mice. This process is *per se* accompanied by massive infiltrates of other immune cells (Kono et al., 2011).

Previous studies have shown that macrophages in the liver are involved in controlling activation and recruitment of neutrophils mainly by secretion of KC (murine IL-8), which in turn induce hepatocyte damage through release of reactive oxygen species and proteases (Chosay et al., 1997). Accordingly, severe liver injury was indeed associated with increased infiltration of neutrophils into the liver of *PF4^-/-^* mice. Thus, unlike in liver fibrosis and other murine models of inflammation (Zaldivar et al., 2010; Hwaiz et al., 2015), where PF4-mediated neutrophil recruitment plays an important pathogenic role, PF4-driven hepatic neutrophil infiltration does not appear to contribute to GalN/LPS-induced liver injury.

Furthermore, PF4 does not seem to be involved in hepatic recruitment of T cell subsets in our experimental setting. Consequently, we hypothesized that macrophages within the liver play a functional role in the phenotype of *PF4^-/-^* mice observed in both models of acute liver injury. However, as PF4 is crucial for monocyte recruitment, survival promotion and pro-inflammatory macrophage differentiation (Scheuerer et al., 2000; von Hundelshausen et al., 2007; Koenen et al., 2009), the question how PF4 exerts its anti-inflammatory effects on macrophages in the liver remains obscure at this point. It has been appreciated that PF4 triggers APC generation (Kowalska et al., 2007). Furthermore, this protein has already been involved in the suppression of the pro-inflammatory immune response of activated monocytes/macrophages *in vitro* and *in vivo* (Stephenson et al., 2006; Kerschen et al., 2007; Cao et al., 2010). Compared to their wild-type counterparts showing a time-dependent increase of APC levels, *PF4^-/-^* mice were indeed unable to generate APC after GalN/LPS treatment, indicating a relevance of PF4 in preventing liver injury in wild-type mice through anti-inflammatory activities of APC. Compared to the results obtained in mouse models also patients with ALF showed increased APC serum levels compared to healthy controls.

Liver resident macrophages, termed Kupffer cells, also contribute to the pro-inflammatory immune response in the GalN/LPS model of liver injury (Antoniades et al., 2008). *In vitro*, PF4 directly repressed the phagocytosis of LPS-stimulated Kupffer cells as well as NO and TNF-a release by these cells. These findings provide the first evidence that platelet-derived PF4 has a crucial role in limiting Kupffer cell activation. Interestingly, activated Kupffer cells were also a source of LPS-stimulated PF4 which likely contributed to the regulation of the pro-inflammatory immune response of Kupffer cells. However, our data cannot exclude other relevant sources in other immune cells, such as T cells, infiltrating monocytes and dendritic cells, after activation (Schaffner et al., 2005; Shi et al., 2014). Additionally PF4 reduced the basal release of TNF-α of *PF4^-/-^* Kupffer cells, *in vitro*. These findings are in line with our *in vivo* data showing increased *TNF-α* mRNA expression in *PF4^-/-^* mice under steady state conditions when compared to wild-type mice. However, the infiltrates of immune cell subsets were not changed in this setting, which could be explained by increased counter-regulatory *IL-10* expression. The data indicates that autocrine PF4 contributes to the immune modulatory function of quiescent Kupffer cells. The phenotype of *PF4^-/-^* mice after acute intoxication could be completely reversed by systemic PF4 administration.

Our *in vitro* and *in vivo* results suggested a potential direct effect of PF4 on distinct features of acute liver injury. Thus, we next investigated the possibility to impede the progression of liver injury *in vivo* by therapeutic PF4 application. Although such a translational study has been conducted in sepsis models (Kowalska et al., 2007), the use of PF4 for modulating acute liver injury has not yet been systematically evaluated. In these experiments, therapy with PF4 indeed reduced liver injury as assessed by histology and TUNEL assay. Notably, the hepatoprotective changes in PF4 treated animals were associated with a decreased infiltration of F4/80^+^ and CD11b^+^ immune cells within the liver alongside with anti-inflammatory cytokine production. As we could not find major differences in neutrophil infiltration between PF4 and vehicle treated mice, the attenuation of liver injury appears to be primarily due to reduced activation of F4/80^+^ immune cells with a subsequent decrease in apoptosis of cells within the liver. Indeed, other drugs mainly targeting Kupffer cells have also been shown to dampen liver injury *in vivo* (Mishra et al., 2013). As anticipated (Kowalska et al., 2007), systemic PF4 also increased plasma levels of APC. Consequently, we hypothesized, that in the liver F4/80^+^ macrophages play a functional role in the phenotype of *PF4^-/-^* mice observed in both models of acute liver injury. We could show that the depletion of these cells with clodrolip in *PF4^-/-^* and wild-type mice prior to GalN/LPS challenge resulted in reduced liver injury which was strongly associated with a decreased pro-inflammatory cytokine profile. Notably, these features were more pronounced in *PF4^-/-^* mice suggesting that PF4 impairs the detrimental response of liver macrophages in acute liver injury.

In summary, we describe an unexpected anti-inflammatory function of PF4 through limiting activation of F4/80^+^ cells and APC-mediated suppression of a pro-inflammatory monocyte/macrophage response. Furthermore, we could associate decreased serum PF4 levels with the progression of acute liver diseases in mouse and man. These findings reveal novel features of this pleiotropic chemokine within the liver and advise further investigations of PF4 as a potential therapeutic approach in acute liver diseases.

## Data Availability

All datasets generated for this study are included in the manuscript and/or the Supplementary Files.

## Ethics Statement

This study was carried out in accordance with the recommendations of ‘the ethics committee of the University Hospital, RWTH Aachen, local IRB permit number EK 166-12’ with written informed consent from all subjects. All subjects gave written informed consent in accordance with the Declaration of Helsinki. The protocol was approved by the ‘ethics committee of the University Hospital, RWTH Aachen.’

This study was carried out in accordance with the recommendations of ‘the Animals Welfare Review Board and by the German legal authorities of North-Rhine Westfalia (LANUV).’ The protocol was approved by the ‘Animals Welfare Review Board and by the German legal authorities of North-Rhine Westfalia (LANUV).’

## Author Contributions

HD: study design, data acquisition, data analysis, and drafting of manuscript. EB: data acquisition, data analysis, and drafting of manuscript. PF: data acquisition. SD: data acquisition and data analysis. RS, MK, and AC: providing samples for analysis. HW, RW, and CT: critical revision of manuscript and intellectual content. M-LB, DK, and HS: study design, fundraising, drafting of manuscript, and study supervision.

## Conflict of Interest Statement

The authors declare that the research was conducted in the absence of any commercial or financial relationships that could be construed as a potential conflict of interest.

## Footnotes

1 http://graphpad.com/quickcalcs/Grubbs1.cfm

## Abbreviations

ALT: alanine aminotransferase
APC: activated Protein C
AST: aspartate aminotransferase
CCl_4_: carbon tetrachloride
ctrl: control
*E. coli*: *Escherichia coli*
GalN: D-galactosamine hydrochloride
LPSs: lipopolysaccharides
NO: nitric oxide
PF4: platelet factor 4.
