# Supplementary material for: Platelet Factor 4 Attenuates Experimental Acute Liver Injury in Mice
Source: Front Physiol. 2019 Mar 26;10:326. doi: 10.3389/fphys.2019.00326 (PMC6444115; doi:10.3389/fphys.2019.00326)

SUPPLEMENTARY FIGURE LEGENDS

Supplementary Table 1: Baseline patient characteristics

Supplementary Figure 1: Quantitative analysis of TUNEL assay showed a significantly increased number of $TUNEL^+$ cells within the liver of $PF4^{-/-}$ mice (**A**). Western blot analysis demonstrated an increase of Caspase-3 activation in $PF4^{-/-}$ mice 2 and 6 hours after GalN/LPS treatment when compared to wild-type mice (**B**). Analysis of F4/80 and CD11b staining revealed a significantly increased number of $F4/80^+$ (**C**) and $CD11b^+$ (**D**) immune cells within the liver of $PF4^{-/-}$ mice compared to wild-type mice 6 hours after GalN/LPS challenge. Flow cytometric analysis of $CD11b^+/F4/80^{low}$ (infiltrating monocytes) and $CD11b^+/F4/80^{high}$ (liver resident macrophages) cells within liver tissue of wild-type and $PF4^{-/-}$ mice 2 and 6 hours after GalN/LPS **(E)**. n=8, *$P < 0.05$, **$P < 0.01$, ***$P < 0.001$.

Supplementary Figure 2: The mRNA expression of the neutrophil chemoattractant *KC* (murine IL-8) was enhanced (**A**) which was associated with a significantly increased number of $Ly6G^+$ neutrophils ($Ly6G^+$ cells (green), nuclei were counterstained with Dapi (blue), magnification x200) (**B**). n=8, *$P < 0.05$, **$P < 0.01$.

Supplementary Figure 3: Flow cytometry analysis showed no differences in frequency and absolute number of $CD4^+$ and $CD8^+$ T cells between $PF4^{-/-}$ and wild-type mice 6 hours after GalN/LPS challenge. n=8.

Supplementary Figure 4: $PF4^{-/-}$ mice showed increased liver injury after CCl_4 challenge compared to wild-type mice as assessed by analysis of H&E stained liver tissues (magnification x100) (**A**). Increased liver injury was reflected by a significant increase of $TUNEL^+$ cells ($TUNEL^+$ cells (green), nuclei were counterstained with Dapi (blue), magnification x200) (**B**) and the inflammation-associated genes *TGF-β* (**C**) and *TNF-α* (**D**) within the liver. n=8, *$P < 0.05$, **$P < 0.01$.

Supplementary Figure 5: Representative picture of Kupffer cells stimulated with DsRed-expressing E. coli and LPS (magnification x200) (**A**). Expression of PF4 was confirmed in primary wild-type Kupffer cells (ctrl) by RT-qPCR, which was increased after LPS stimulation and completely blunted in untreated (ctrl) and LPS treated $PF4^{-/-}$ Kupffer cells. β-actin was used as reference gene (**B**).

Supplementary Figure 6: In the GalN/LPS model, analysis of TUNEL assay revealed a significantly decreased number of $TUNEL^+$ cells within the liver of recombinant mouse PF4 (rmPF4) treated mice when compared to vehicle treated mice (**A**). Western blot analysis showed decreased Caspase-3 activation in PF4 treated mice (**B**). Moreover, PF4 therapy significantly decreased the number of $F4/80^+$ (**C**) and $CD11b^+$ (**D**) immune cells in these mice. n=6, *$P < 0.05$.

Supplementary Figure 7: Deletion of $F4/80^+$ macrophages in the liver could already be achieved by administration of a single dose clodrolip compared to animals injected with

empty liposomes as control (F4/80^+ cells (green), nuclei were counterstained with Dapi (blue), magnification x200).

Supplementary Table 1

Clinical and demographic characteristics of the patients	
Age (y)	40.86 ± 3.78
Sex (F/M, no)	14/8
Drug toxicity (no)	14 (22)
GOT (U/L)	1269.45 ± 491.68
GPT (U/L)	1353.61 ± 474.09
AP (U/L)	134.33 ± 19.18
GGT (U/L)	188.45 ± 44.19
LDH (U/L)	832.05 ± 241.62
GLDH (U/L)	391.98 ± 243.27
Billi (mg/dL)	10.26 ± 2.2
TPZ (%)	55.3 ± 6.36
INR	1.65 ± 0.16
aPTT (s)	39.45 ± 3.26

Supplementary Figure 1

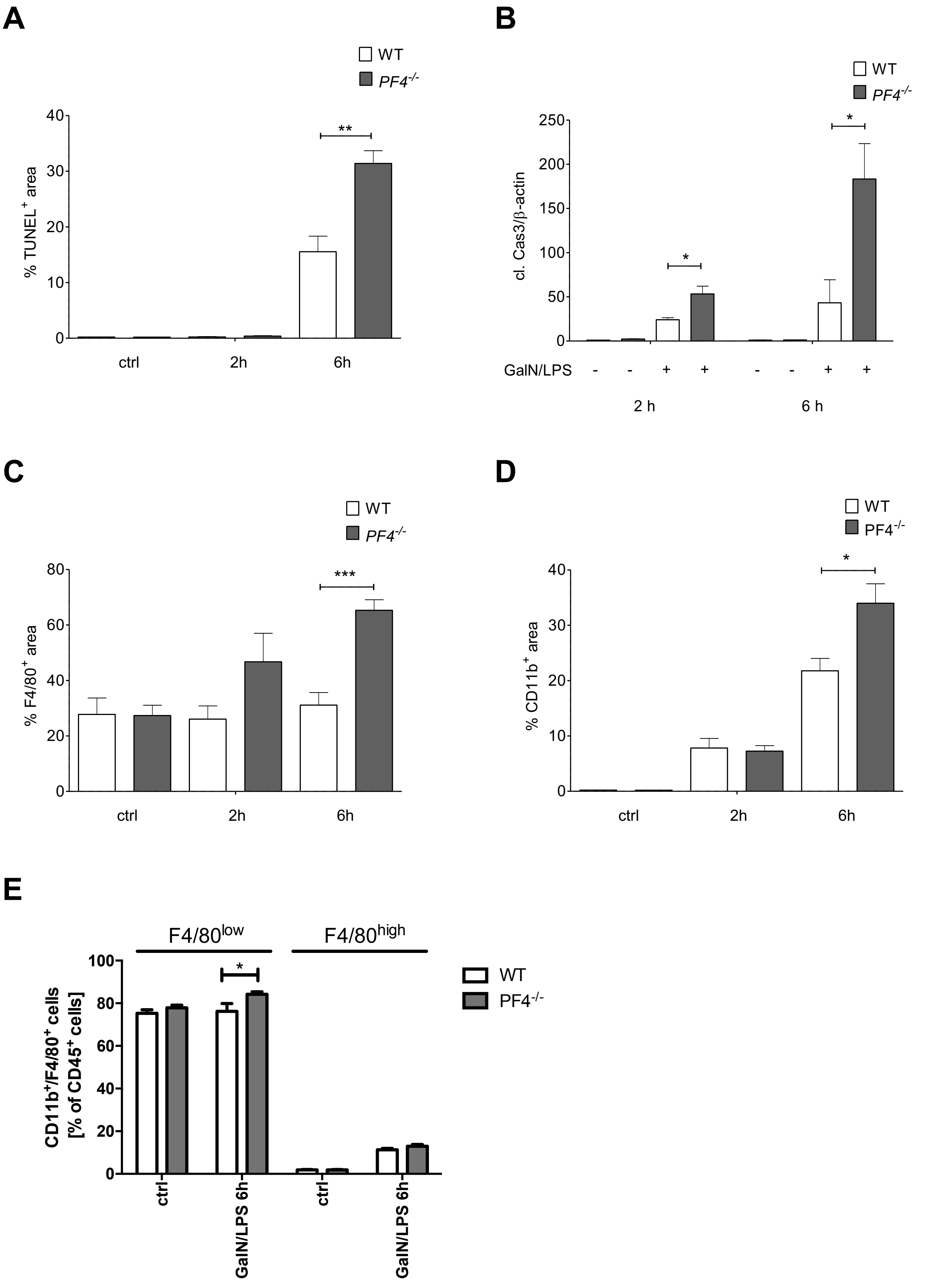

Supplementary Figure 2

A

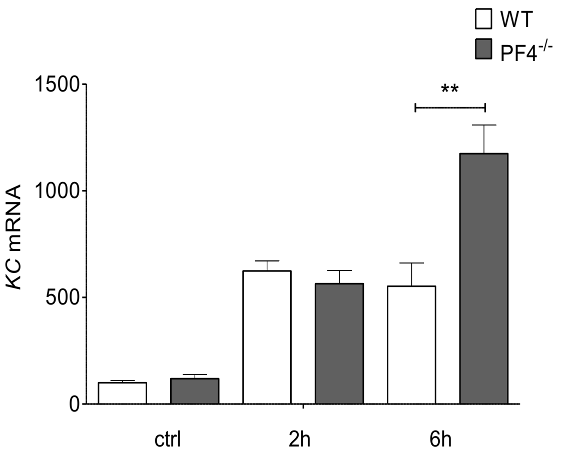

B

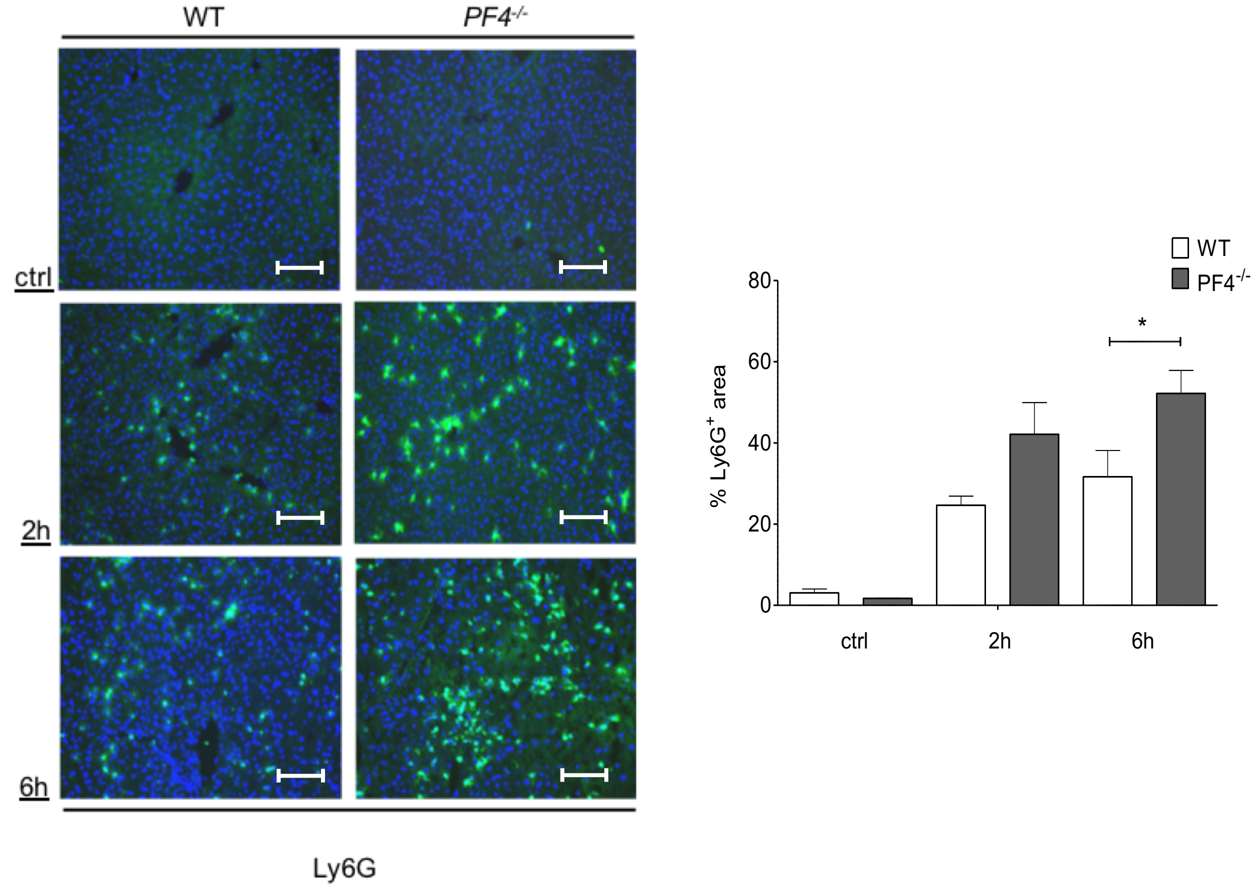

Supplementary Figure 3

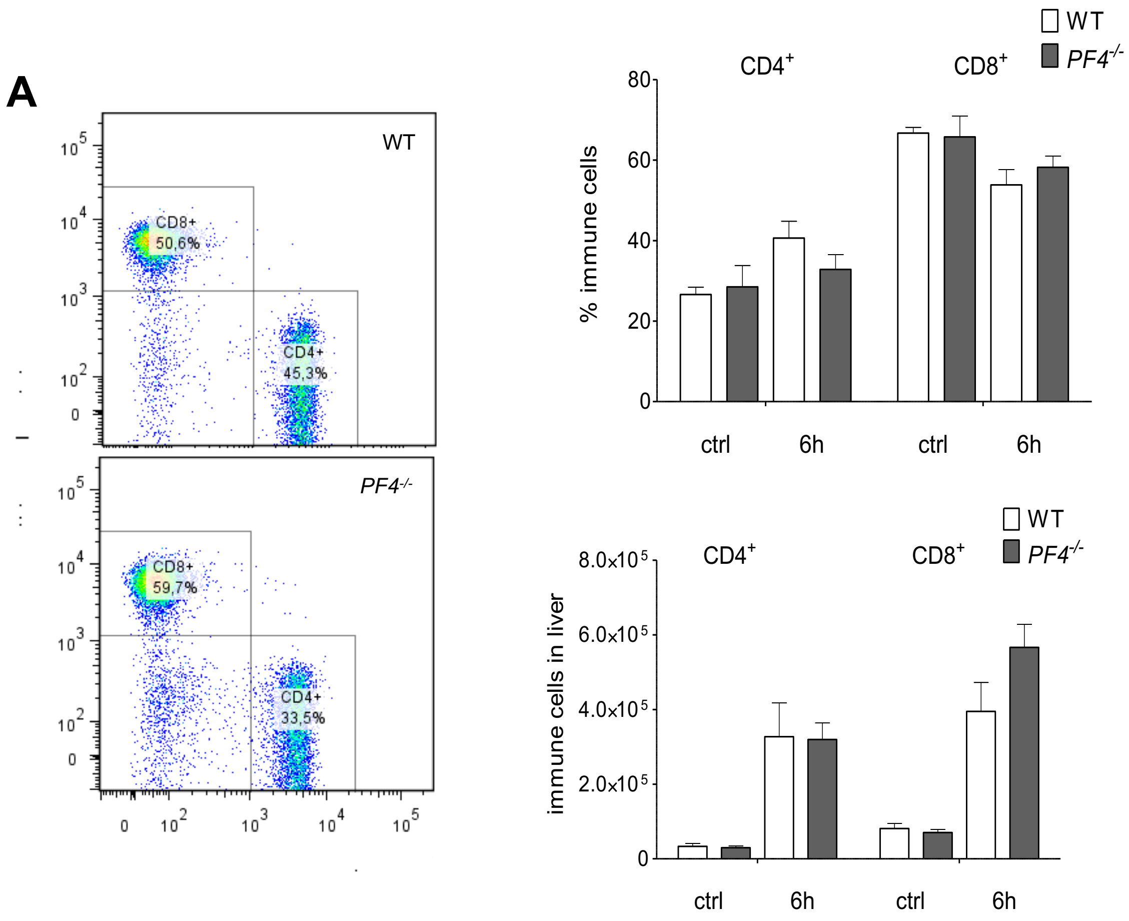

Supplementary Figure 4

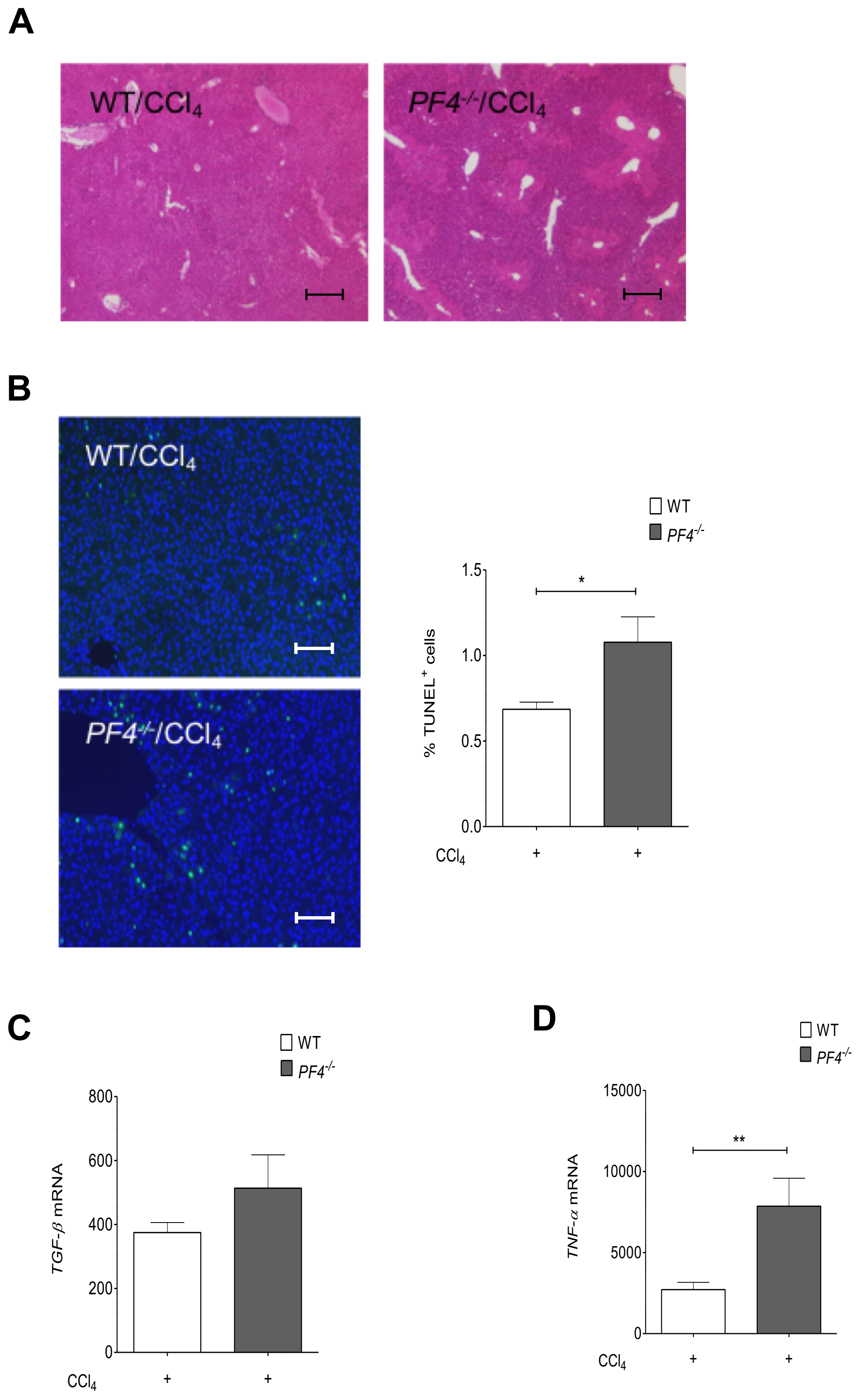

Supplementary Figure 5

A

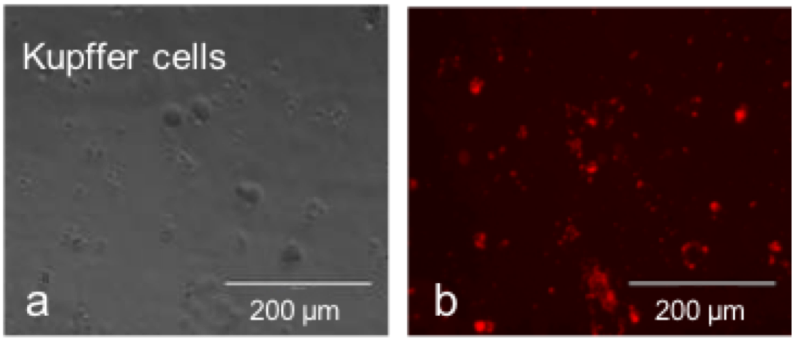

B

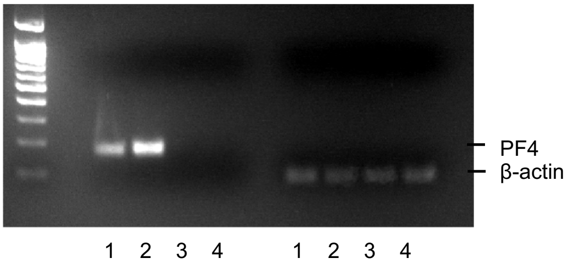

1 WT Kupffer cells/ctrl
2 WT Kupffer cells/LPS
3 *PF4*$^{-/-}$ Kupffer cells/ctrl
4 *PF4*$^{-/-}$ Kupffer cells/LPS

Supplementary Figure 6

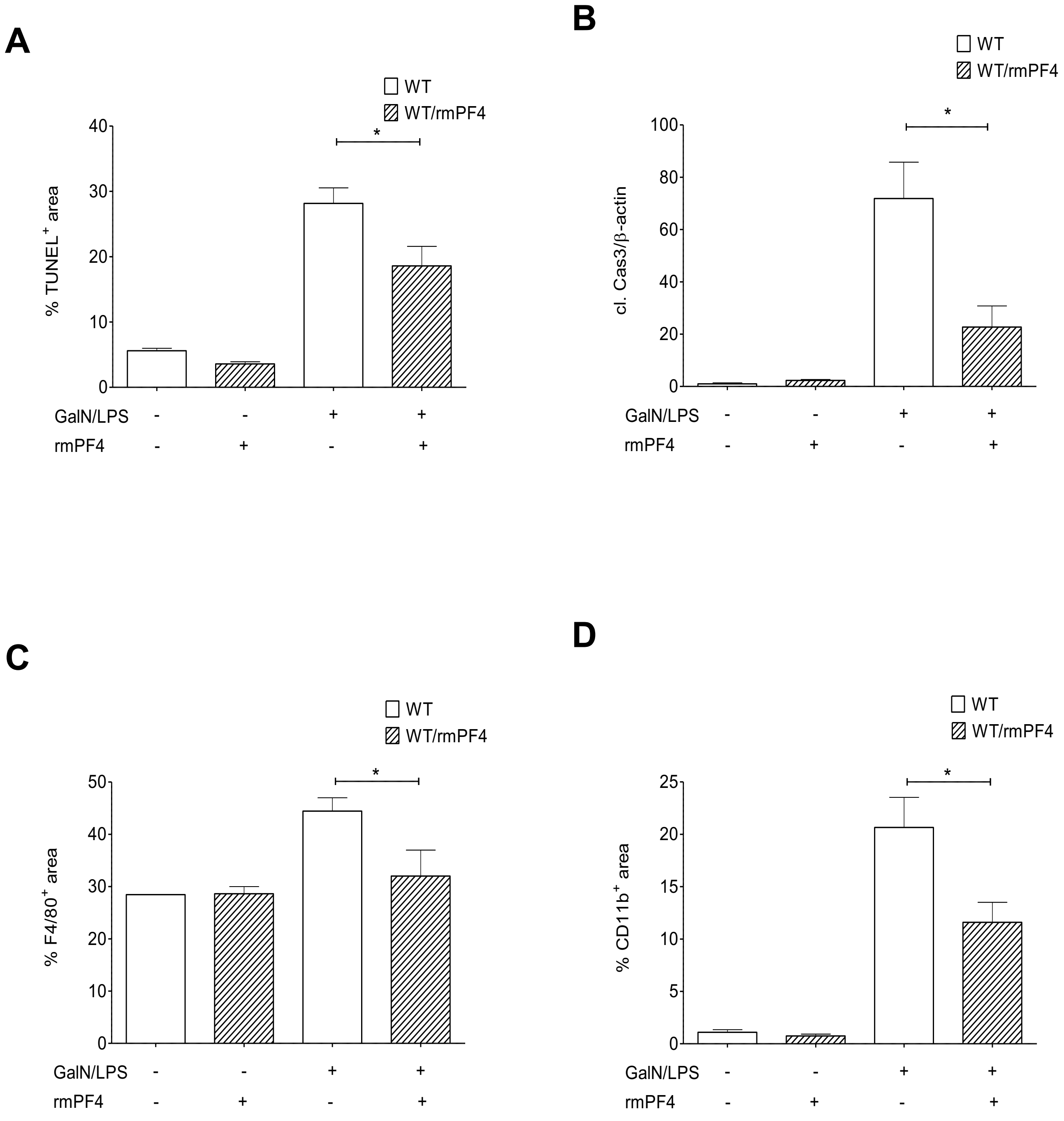

Supplementary Figure 7

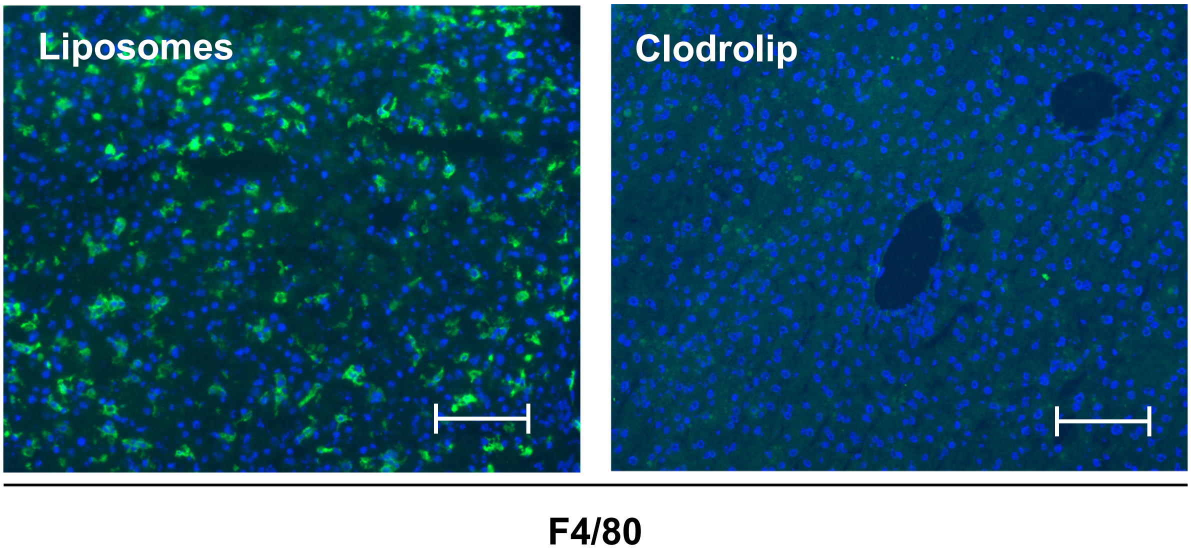

Supplement: Supplementary file 1 [file Presentation_1.pdf]
